# Cigarette Smoking Is Associated with a Lower Prevalence of Newly Diagnosed Diabetes Screened by OGTT than Non-Smoking in Chinese Men with Normal Weight

**DOI:** 10.1371/journal.pone.0149234

**Published:** 2016-03-08

**Authors:** Xuhong Hou, Jieyuzhen Qiu, Peizhu Chen, Jun Lu, Xiaojing Ma, Juming Lu, Jianping Weng, Linong Ji, Zhongyan Shan, Jie Liu, Haoming Tian, Qiuhe Ji, Dalong Zhu, Jiapu Ge, Lixiang Lin, Li Chen, Xiaohui Guo, Zhigang Zhao, Qiang Li, Zhiguang Zhou, Wenying Yang, Weiping Jia, China National Diabetes Metabolic Disorders Study Group

**Affiliations:** 1 Department of Endocrinology and Metabolism, Shanghai Jiao Tong University Affiliated Sixth People’s Hospital, Shanghai Diabetes Institute, Shanghai Clinical Center for Diabetes, Shanghai, China; 2 Department of Endocrinology and Metabolism, Chinese People’s Liberation Army General Hospital, Beijing, China; 3 Department of Endocrinology and Metabolism, The Third Affiliated Hospital of Sun Yat-sen University, Guangzhou, China; 4 Department of Endocrinology and Metabolism, Peking University People’s Hospital, Beijing, China; 5 Department of Endocrinology and Metabolism, The First Affiliated Hospital of China Medical University, Shenyang, Liaoning, China; 6 Department of Endocrinology and Metabolism, Shanxi Provincial People’s Hospital, Taiyuan, Shanxi, China; 7 Department of Endocrinology and Metabolism, West China Hospital, Sichuan University, Chengdu, Sichuan, China; 8 Department of Endocrinology and Metabolism, Xijing Hospital, The Fourth Military Medical University, Xi’an, Shaanxi, China; 9 Department of Endocrinology and Metabolism, The Affiliated Drum Tower Hospital of Nanjing University Medical School, Nanjing, Jiangsu, China; 10 Department of Endocrinology and Metabolism, People’s Hospital of Xinjiang Uygur Autonomous Region, Urumqi, Xinjiang, China; 11 Department of Endocrinology and Metabolism, Fujian Provincial Hospital, Fuzhou, Fujian, China; 12 Department of Endocrinology and Metabolism, Qilu Hospital of Shandong University, Jinan, Shandong, China; 13 Department of Endocrinology and Metabolism, Peking University First Hospital, Beijing, China; 14 Department of Endocrinology and Metabolism, Henan Provincial People’s Hospital, Zhengzhou, Henan, China; 15 Department of Endocrinology and Metabolism, The Second Affiliated Hospital of Harbin Medical University, Harbin, Heilongjiang, China; 16 Department of Endocrinology and Metabolism, The Second Xiangya Hospital of Central South University, Changsha, Hunan, China; 17 Department of Endocrinology and Metabolism, China-Japan Friendship Hospital, Beijing, China; Medical University Innsbruck, AUSTRIA

## Abstract

Different studies have produced conflicting results regarding the association between smoking and diabetes mellitus, and detailed analysis of this issue in Chinese males based on nationwide samples is lacking. We explored the association between cigarette smoking and newly-diagnosed diabetes mellitus (NDM) in Chinese males using a population-based case-control analysis; 16,286 male participants without previously diagnosed diabetes were included. Prediabetes and NDM were diagnosed using the oral glucose tolerance test. The cohort included 6,913 non-smokers (42.4%), 1,479 ex-smokers (9.1%) and 7,894 current smokers (48.5%). Age-adjusted glucose concentrations (mmol/L) were significantly lower at fasting and 120 min in current smokers than non-smokers (5.25 vs. 5.30, 6.46 vs. 6.55, respectively, both *P* < 0.01). After adjustment for demographic and behavioral variables (age, region, alcohol consumption status, physical activity, education, and family history of diabetes), logistic regression revealed significant negative associations between smoking and NDM in males of a normal weight (BMI < 25 kg/m^2^: adjusted odds ratio [AOR] = 0.75, *P* = 0.007; waist circumference < 90 cm: AOR = 0.71, *P* = 0.001) and males living in southern China (AOR = 0.75, *P* = 0.009), but not in males who were overweight/obese, males with central obesity, or males living in northern China. Compared to non-smokers, current smokers were less likely to be centrally obese or have elevated BP (AOR: 0.82 and 0.74, both *P* < 0.05), and heavy smokers (≥ 20 pack-years) were less likely to have elevated TG (AOR = 0.84, *P* = 0.012) among males of a normal weight. There were no significant associations between quitting smoking and metabolic disorders either among males of a normal weight or males who were overweight/obese. In conclusion, smokers have a lower likelihood of NDM than non-smokers among Chinese males with a lower BMI/smaller waist.

## Introduction

Despite substantial progress in global tobacco control, there are about 1.1 billion smokers worldwide, and 300 million alone in China [1]. Simultaneously, there has been an alarmingly rapid increase in the prevalence of diabetes in China [2] and the rest of the world [3]. Does the diabetes epidemic relate to smoking exposure, and can it be partly attributed to smoking exposure? The exact mechanisms of the effect of smoking on diabetes are not yet fully understood. Although most epidemiological studies support the premise that cigarette smoking is a preventable cause of diabetes [4–6], a few studies, which include prospective population-based studies, have identified no association [7–9] or even reported a protective effect of smoking against diabetes [10, 11]. Some studies have demonstrated a complicated association between cigarette smoking and diabetes that is modified by body mass index (BMI) [5, 11]. A recent meta-analysis of 14 studies concluded that current smokers had comparable fasting plasma glucose (FPG) and lower 2-h postprandial plasma glucose (2-h PG) than never-smokers [12], which indicates that newly-diagnosed diabetes mellitus (NDM), as detected by screening via the oral glucose tolerance test (OGTT), may be more frequent in non-smokers than in current smokers.

The first paper on the prevalence of diabetes based on the China National Diabetes and Metabolic Disorders Study (CNDMDS) dataset stated that “Patients with previously diagnosed diabetes, as compared with those with undiagnosed diabetes, were less likely to smoke cigarettes” [2]. Another large-scale population-based survey in Chinese adults also reported that current cigarette smoking was associated with a lower likelihood of diabetes (adjusted odds ratio [AOR] = 0.84, *P* < 0.001], which was defined as a previous diagnosis of diabetes, elevated FPG (≥ 7.0 mmol/L), elevated 2-h PG (≥ 11.1 mmol/L), or elevated glycated Hemoglobin A_1c_ (HbA_1c_; ≥ 6.5%) [13]. It is not yet clear if this relationship also applies to individuals with NDM. To date, there have been no large-scale nationwide reports intensively discussing this issue in detail in Chinese males, who represent the overwhelming majority of smokers in the Chinese population.

In this paper, we aimed to assess the association between cigarette smoking and NDM as screened by the OGTT among Chinese males using the population-based CNDMDS dataset [2].

## Materials and Methods

### Study design and population

We conducted a case-control analysis based on the CNDMDS dataset, a representative prevalence survey of diabetes in Chinese adults conducted between June 2007 and May 2008 [2]. The multi-stage sampling process has been described previously [2] and is introduced briefly here.

According to geographic region, degree of urbanization and economic development status, 11 provinces, one autonomous region and two municipalities were selected, and cities and counties were selected from these provinces. Then, 152 urban street districts and 112 rural villages were selected from city districts and rural townships. Individuals who were ≥ 20 years old and had lived at their current residence for 5 years or more were eligible to participate. Target populations were stratified by sex and age group and sampled on the basis of the sex- and age- structure of the Chinese population in 2006.

A total of 54,240 residents were selected and invited to participate in this survey; 47,325 individuals participated. Complete data on demographic information, FPG and 2-h PG was collected for 46,239 participants, of whom 18,419 were male. After excluding 894 male subjects with previously diagnosed diabetes, 594 male subjects without complete smoking information and 645 male subjects with a BMI < 18.5 kg/m^2^, we finally included a total of 16,286 male subjects aged 20–98 years without known diabetes but with complete smoking information (including 119 current smokers who were relapsed smokers) in this study.

### Ethics Statement

The study program was reviewed and approved by the Clinical Research Ethics Committee of China-Japan Friendship Hospital (No. 2007–026). Each subject provided written informed consent prior to data collection.

### Blood sample collection and laboratory examination

Subjects without a prior diagnosis of diabetes mellitus (DM) underwent an OGTT. Subjects fasted overnight for at least 10 h before venous blood samples were drawn at 0, 30 and 120 min following oral ingestion of 75 g glucose. Plasma glucose, serum cholesterol, and triglyceride (TG) levels were measured using enzymatic assays. A radioimmunoassay was used to measure serum insulin levels.

### Estimation of beta cell function and insulin sensitivity

Homeostasis Model Assessment-Insulin Resistance (HOMA-IR) was calculated as Ins_0_ × Gluc_0_/22.5 [14]. Peripheral insulin sensitivity index (ISI_Gutt_) was calculated as: M/MG/lgMI, where M = 75000/120 + [Gluc_0_ − Gluc_120_] × 180 × 0.19 × weight /120, MG = (Gluc_0_ + Gluc_120_)/2, and lgMI = lg (Ins_0_/2 + Ins_120_/2) [15]. Gluc_0_, Ins_0_, Gluc_120_, and Ins_120_ refer to the glucose and insulin levels at 0 min and 120 min, respectively.

### Physical examination

Blood pressure (BP), body weight and height were measured according to standard protocols [16]. BMI was derived by dividing the weight by height squared (kg/m^2^). Waist circumference was measured at the horizontal plane between the inferior costal margin and the iliac crest on the mid-axillary line and waist-to-height ratio was calculated by dividing waist size by height.

### Questionnaire

Subjects were asked whether they currently smoked regularly or had ever smoked. Regular smokers were asked at what age they began smoking and the average number of cigarettes smoked per day; ex-smokers were asked at what age they stopped smoking. Those who had never smoked or who smoked occasionally were considered non-smokers; current smokers smoked at least one cigarette per day and had smoked for at least one year; ex-smokers had regularly smoked previously but had stopped smoking for at least one year. The duration of smoking was calculated for current smokers. Pack-years were calculated as follows: packs (one pack = 20 cigarettes) smoked per day × years of smoking. For current smokers, smoking intensity was assessed according to the number of cigarettes per day (light: 1–19; heavy: ≥ 20) or pack-years (light: 1–19; heavy: ≥ 20).

Information regarding alcohol consumption status, physical activity, education, and family history of diabetes in first-degree relatives was also obtained, as previously described [17].

### Diagnostic criteria for metabolic disorders

The World Health Organization (WHO) report (1999) defined NDM as FPG ≥ 7.0 mmol/L or 2-h PG ≥ 11.1 mmol/L with no previous diagnosis of diabetes; prediabetes as FPG ≥ 6.1 mmol/L and < 7.0 mmol/L, or 2-h PG ≥ 7.8 mmol/L and < 11.1 mmol/L; and normal glucose regulation (NGR) as FPG < 6.1 mmol/L and 2-h PG < 7.8 mmol/L [18].

According to the criteria established by the Chinese Joint Committee for Developing Chinese Guidelines on Prevention and Treatment of Dyslipidemia in Adults (JCDCG), metabolic syndrome (MetS) was defined as ≥ 3 of the following abnormalities: central obesity (waist circumference ≥ 90 cm); elevated TG (TG ≥ 1.7 mmol/L, or specific treatment for this lipid abnormality); reduced high density lipoprotein cholesterol (HDL-C < 1.03 mmol/l); elevated BP (BP ≥ 130/85 mmHg or previously diagnosed hypertension); or elevated plasma glucose (FPG ≥ 6.1 mmol/L, 2-h PG ≥ 7.8 mmol/L, or previously diagnosed DM) [19]. In addition, a BMI < 18.5 kg/m^2^ was defined as underweight and a BMI ≥ 25 kg/m^2^ as overweight or obese using the WHO standards [20].

### Statistical analysis

Continuous variables are expressed as the mean (standard deviation [SD]), with the exception of the indices of insulin, insulin resistance, and insulin sensitivity, which are presented as the median (interquartile range [IQR]); categorical variables are presented as frequency (percentage). We first conducted analysis of variance (ANOVA), analysis of covariance (ANCOVA), or the Kruskal–Wallis test (as appropriate) to draw an overall *P*-value of comparison of three groups, and then do the appropriate post hoc pairwise comparisons. Except for mean age, which was examined using ANOVA, we used ANCOVA to examine the differences in the mean values between two groups after adjusting for age, examined the differences in the median values using the Mann-Whitney U-test, and compared differences in the proportions using the chi-square test. The Bonferroni correction *P* value level (*P* < 0.016 [two-sided]) was used for multiple comparisons when using the latter two methods.

Multinomial multivariable logistic regression was conducted to assess the association between smoking exposure and glycemic regulation status (NGR, prediabetes, NDM) using the Enter method. Multivariable binary logistic regression was used to assess the association of smoking exposure with diabetes and other metabolic disorders (yes/no). For these analyses, the independent variables were the smoking exposure indices with or without the adjustment variables. The adjustment variables included demographic and behavioral variables (age, family history of diabetes, residence [southern/northern, urban/rural], education, alcohol consumption status, physical activity), and metabolic variables (waist circumference, TG, HDL-C, systolic blood pressure [SBP]). The crude OR (COR), AOR and their 95% CIs were reported.

In addition, multinomial logistic regression analysis was also conducted to examine the interactions between smoking status and BMI categories, or waist categories on glycemic regulation status (NGR, prediabetes, NDM) by including products (interactions) of different sets of two variables.

Statistical analyses were performed using SPSS version 21.0 (SPSS Inc., Chicago, IL, USA); *P* < 0.05 (two-sided) was considered statistically significant.

## Results

A total of 16,286 male subjects without previously diagnosed diabetes, comprising 6,913 non-smokers (42.4%), 1,479 ex-smokers (9.1%) and 7,894 current smokers (48.5%) were included in this study. Among the total population, 1,246 new cases of diabetes were diagnosed. Of the current smokers, 52.8% smoked ≥ 20 cigarettes daily, 61.2% had smoked for ≥ 20 years and 44.9% had smoked ≥ 20 pack-years (Data not shown in Tables).

### Demographic and clinical characteristics according to smoking status (Table 1)

The average age of non-, ex-, and current smokers was 43.1 years, 52.4 years, and 44.4 years, respectively (all *P* < 0.001). Compared to non-smokers, both current and ex-smokers had significantly higher 30-min postprandial plasma glucose (30-min PG), and TG, and comparable levels of total cholesterol (TC) and HDL-C. In contrast to non-smokers, current smokers had significantly lower FPG, 2-h PG, fasting plasma insulin (FINS), 2-h postprandial plasma insulin (2-h INS), HOMA-IR, and SBP, and higher ISI_Gutt_ and larger waist circumference. Waist-to-height ratio, BMI, low density lipoprotein cholesterol (LDL-C), and diastolic blood pressure (DBP) were not significantly different between current smokers and non-smokers. Ex-smokers had a significantly higher waist-to-height ratio, BMI, larger waist circumference, and elevated SBP than non-smokers. Current smokers had higher frequencies of alcohol consumption and family histories of diabetes, and were less likely to participate in physical activity than non-smokers (Table 1).

**Table 1:** Demographic and clinical characteristics of the subjects according to smoking status.

Characteristics	Non-smoker (I)	Ex-smoker (II)	Current smoker (III)	*P*-value	*P*-value	*P*-value
	(*n* = 6,913)	(*n* = 1,479)	(n = 7,894)	Overall	(II vs. I)	(III vs. I)
Age (years)	43.1 (14.9)	52.4 (13.6)	44.4 (12.6)	< 0.001	< 0.001	< 0.001
Waist circumference (cm)	85.3 (10.3)	88.6 (9.5)	85.8 (10.2)	< 0.001	< 0.001	0.038
Waist-to-height ratio	0.510 (0.061)	0.528 (0.056)	0.509 (0.059)	< 0.001	< 0.001	0.065
BMI (kg/m^2^)	24.7 (3.5)	25.4 (3.3)	24.6 (3.5)	< 0.001	< 0.001	0.098
FPG (mmol/L)	5.30 (1.11)	5.49 (1.24)	5.25 (1.18)	< 0.001	0.037	< 0.001
30-min PG (mmol/L)	8.86 (2.35)	9.37 (2.51)	9.06 (2.35)	< 0.001	0.002	< 0.001
2-h PG (mmol/L)	6.55 (2.73)	7.18 (3.19)	6.46 (2.85)	< 0.001	0.019	0.001
TC (mmol/L)	4.71 (0.98)	4.86 (0.96)	4.72 (0.95)	0.274	–	–
TG (mmol/L)	1.65 (1.16)	1.82 (1.29)	1.82 (1.40)	< 0.001	< 0.001	< 0.001
HDL-C (mmol/L)	1.27 (0.33)	1.28 (0.34)	1.26 (0.33)	0.726	–	–
LDL-C (mmol/L)	2.77 (0.82)	2.91 (0.84)	2.78 (0.84)	0.076	–	–
SBP (mmHg)	124.9 (18.0)	130.5 (19.6)	124.5 (17.9)	< 0.001	< 0.001	0.002
DBP (mmHg)	80.5 (11.3)	82.7 (11.8)	80.7 (11.4)	0.016	0.005	0.701
FINS (μIU/mL)	7.43 (5.34–10.33)	7.68 (5.40–10.57)	6.78 (4.94–9.68)	< 0.001	0.109^a^	< 0.001^a^
30-min INS (μIU/mL)	35.88 (20.93–60.11)	36.46 (20.35–64.34)	32.93 (18.66–56.24)	< 0.001	0.769^a^	< 0.001^a^
2-h INS (μIU/mL)	25.38 (13.97–44.68)	28.73 (16.34–51.13)	22.37 (12.86–40.94)	< 0.001	< 0.001^a^	< 0.001^a^
HOMA-IR (mU·mmol /L^2^)	1.71 (1.20–2.48)	1.83 (1.25–2.59)	1.53 (1.07–2.27)	< 0.001	0.007^a^	< 0.001^a^
ISI_Gutt_ [mg·L^2^/ (mmol·mU·min)]	88.35 (65.78–117.01)	78.86 (57.17–106.10)	93.48 (68.87–124.12)	< 0.001	< 0.001^a^	< 0.001^a^
Living in northern China (%)	4,426 (64.0)	1,004 (67.9)	4,957 (62.8)	0.001	0.005	0.121
Educational level				< 0.001	< 0.001^a^	< 0.001^a^
Low (%)	1,310 (19.1)	296 (20.2)	1,348 (17.2)		–	–
Medium (%)	3,281 (47.7)	834 (56.9)	4,855 (61.9)		–	–
High (%)	2,283 (33.2)	337 (23.0)	1,646 (21.0)		–	–
Current drinker (%)	1,937 (28.0)	713 (48.6)	4,601 (58.6)	< 0.001	< 0.001^a^	< 0.001^a^
Physical activity (%)	2,553 (37.0)	701 (47.6)	2,449 (31.2)	< 0.001	< 0.001^a^	< 0.001^a^
Family history of DM (%)	692 (10.0)	183 (12.4)	1,016 (12.9)	< 0.001	0.016^a^	< 0.001^a^
Glucose regulation status				< 0.001	< 0.001^a^	0.343^a^
NGR (%)	5,296 (76.6)	1,023 (69.2)	6,117 (77.5)		–	–
Prediabetes (%)	1,096 (15.9)	283 (19.1)	1,225 (15.5)		–	–
DM (%)	521 (7.5)	173 (11.7)	552 (7.0)		–	–

Data are mean (SD), median (IQR), or *n* (percentage).

We first conducted ANOVA, ANCOVA, or the Kruskal–Wallis test (as appropriate) to drew an overall *P*-value of comparison of three groups and then do the appropriate post hoc pairwise comparisons. Differences in means between the two groups were examined after adjusting for age using ANCOVA, aside from age which was assessed using ANOVA; differences in medians were examined using the Mann-Whitney *U*-test; differences in proportions were tested using the Chi-square test.

^a^The Bonferroni correction was applied for multiple testing (*P* < 0.016 [two-sided] was statistically significant) when using the Mann-Whitney U-test and the chi-square test.

Abbreviations: 2-h INS: 2-h postprandial insulin; 2-h PG: 2-h postprandial plasma glucose; ANCOVA: analysis of covariance; ANOVA: analysis of variance; BMI: body mass index; DM: diabetes mellitus; DBP: diastolic blood pressure; FINS: fasting insulin; FPG: fasting plasma glucose; HDL-C: high-density lipoprotein cholesterol; HOMA-IR: Homeostasis Model Assessment-Insulin Resistance; ISI_Gutt_: peripheral insulin sensitivity index; LDL-C: low-density lipoprotein cholesterol; NGR: normal glucose regulation; SBP: systolic blood pressure; 30-min INS: 30-min postprandial insulin; 30-min PG: 30-min postprandial plasma glucose; TC: total cholesterol; TG: triglycerides.

Further analysis of age-adjusted means showed that compared to non-smokers, light smokers (1–19 pack-years) had a larger waist circumference (85.9 cm vs. 85.4 cm, *P* = 0.015), but heavy smokers (≥ 20 pack-years) had a similar waist circumference to non-smokers (85.5 cm vs. 85.4 cm, *P* = 0.450). Additionally, heavy smokers had a slightly lower BMI than non-smokers or light smokers (24.5 kg/m^2^ vs. 24.7 kg/m^2^ or 24.7 kg/m^2^, both *P* < 0.05; data not shown in Tables).

### Associations of smoking exposure with prediabetes and diabetes (Table 2)

In Model 2, after adjustment for demographic and behavioral variables, there was a significant but weak negative association of prediabetes with current smoking; the corresponding AOR was 0.91 (*P* < 0.05). However, this significant association disappeared after adjustment for metabolic variables in addition to the potential confounders in Model 2 (Model 3).

**Table 2:** Associations of smoking exposure with prediabetes and NDM.

Subgroup	Model 1^a^		Model 2^b^		Model 3^c^	
	Crude OR	*P*-value	Adjusted OR	*P*-value	Adjusted OR	*P*-value
**Prediabetes (*n* = 2,604)**						
Smoking status						
Non-smoker	1 (Reference)		1 (Reference)		1 (Reference)	
Ex-smoker	1.34 (1.15–1.55)	< 0.001	0.95 (0.81–1.11)	0.507	0.89 (0.76–1.05)	0.163
Current smoker	0.97 (0.88–1.06)	0.472	0.91 (0.82–0.999)	0.049	0.94 (0.85–1.04)	0.219
Daily consumption						
Non-smoker	1 (Reference)		1 (Reference)		1 (Reference)	
1–19 cigarettes/day	0.93 (0.83–1.04)	0.190	0.91 (0.81–1.03)	0.134	0.96 (0.85–1.08)	0.482
≥ 20 cigarettes/day	1.00 (0.90–1.12)	0.948	0.89 (0.80–1.00)	0.051	0.92 (0.82–1.04)	0.175
Pack-years						
Non-smoker	1 (Reference)		1 (Reference)		1 (Reference)	
1–19 pack-years	0.78 (0.70–0.87)	< 0.001	0.89 (0.79–1.00)	0.055	0.92 (0.81–1.04)	0.171
≥ 20 pack-years	1.22 (1.10–1.36)	< 0.001	0.91 (0.81–1.03)	0.132	0.96 (0.85–1.08)	0.493
**NDM (*n* = 1,246)**						
Smoking status						
Non-smoker	1 (Reference)		1 (Reference)		1 (Reference)	
Ex-smoker	1.72 (1.43–2.07)	< 0.001	1.11 (0.92–1.35)	0.278	0.98 (0.80–1.20)	0.858
Current smoker	0.92 (0.81–1.04)	0.177	0.80 (0.70–0.92)	0.002	0.81 (0.70–0.93)	0.004
Daily consumption						
Non-smoker	1 (Reference)		1 (Reference)		1 (Reference)	
1–19 cigarettes/day	0.88 (0.75–1.02)	0.097	0.81 (0.69–0.96)	0.014	0.84 (0.71–1.00)	0.051
≥ 20 cigarettes/day	0.96 (0.82–1.11)	0.549	0.80 (0.68–0.94)	0.007	0.80 (0.68–0.94)	0.009
Pack-years						
Non-smoker	1 (Reference)		1 (Reference)		1 (Reference)	
1–19 pack-years	0.71 (0.61–0.83)	< 0.001	0.80 (0.67–0.94)	0.008	0.80 (0.67–0.95)	0.011
≥ 20 pack-years	1.20 (1.03–1.39)	0.017	0.82 (0.69–0.96)	0.014	0.84 (0.71–0.99)	0.042

^a^Model 1: Without adjusted variables

^b^Model 2: Adjusted for area of residence (northern/southern, urban/rural), age, alcohol consumption status, physical activity, education level, family history of diabetes

^c^Model 3: Adjusted for variables in Model 2 plus waist circumference, TG, HDL-C and SBP

Abbreviations: HDL-C: high-density lipoprotein cholesterol; NDM: newly-diagnosed diabetes mellitus; OR: odds ratio; SBP: systolic blood pressure; TG: triglycerides.

In Model 2, there were negative associations between the likelihood of NDM and smoking exposure (smoking status, smoking amount), with AORs ranging from 0.80–0.82 for current smokers with different levels of daily consumption or pack-years (all *P* < 0.05). In Model 3, there was no change in the strength of the negative association between smoking exposure and likelihood of diabetes, even after adjusting for metabolic indices, except for smokers with light daily consumption.

### Interaction analysis between smoking status and BMI categories, or waist categories on NDM (Table 3)

After adjusting for potential confounders, the association of NDM with an interaction term (between smoking status and BMI categories) was significant (*P* < 0.001). With the subpopulations exposed to neither factor (non-smoking in males with a normal weight) as a reference, the corresponding AORs were 0.74 for smoking alone (in males with a normal weight), 2.08 for overweight/obesity alone (in male non-smokers), and 1.90 for the joint effect of both risk factors (smoking and overweight/obesity, i.e., in male smokers who were overweight or obese). Similar trends were generated when assessing the relationship of NDM to an interaction term (between smoking status and waist categories) (*P* < 0.001).

**Table 3:** Interaction between smoking status and BMI categories, or waist categories on prediabetes and NDM.

Interaction terms	*N*	Prediabetes		NDM	
		Adjusted OR (95% CI) ^a^	*P*	Adjusted OR (95% CI) ^a^	*P*
Smoking status and BMI category					
No smoking & normal weight	4,042	1	< 0.001	1	< 0.001
No smoking & overweight/obesity	2,871	1.74 (1.52–2.00)	< 0.001	2.08 (1.72–2.51)	< 0.001
Quitting smoking & normal weight	702	0.84 (0.66–1.07)	0.154	0.93 (0.67–1.29)	0.658
Quitting smoking & Overweight/obesity	777	1.71 (1.39–2.09)	< 0.001	2.41 (1.87–3.12)	< 0.001
Smoking & normal weight	4,627	0.84 (0.74–0.97)	0.014	0.74 (0.60–0.90)	0.003
Smoking & overweight/obesity	3,267	1.77 (1.54–2.02)	< 0.001	1.90 (1.57–2.29)	< 0.001
Smoking status and waist circumference category					
No smoking & normal weight	4,565	1	< 0.001	1	< 0.001
No smoking & central obesity	2,336	1.64 (1.43–1.89)	< 0.001	2.02 (1.67–2.43)	< 0.001
Quitting smoking & normal weight	794	0.92 (0.74–1.15)	0.474	0.81 (0.59–1.12)	0.200
Quitting smoking & central obesity	683	1.54 (1.24–1.91)	< 0.001	2.57 (1.99–3.31)	< 0.001
Smoking & normal weight	5,114	0.85 (0.75–0.96)	0.011	0.70 (0.57–0.84)	< 0.001
Smoking & central obesity	2,764	1.68 (1.47–1.93)	< 0.001	1.96(1.62–2.36)	< 0.001

^a^Adjusted for area of residence (northern/southern, urban/rural), age, alcohol consumption status, physical activity, education level, family history of diabetes

Abbreviations: NDM: newly-diagnosed diabetes mellitus; OR: odds ratio.

### Association between smoking exposure and NDM according to BMI, waist circumference, or region strata (Fig 1)

**Fig 1:**
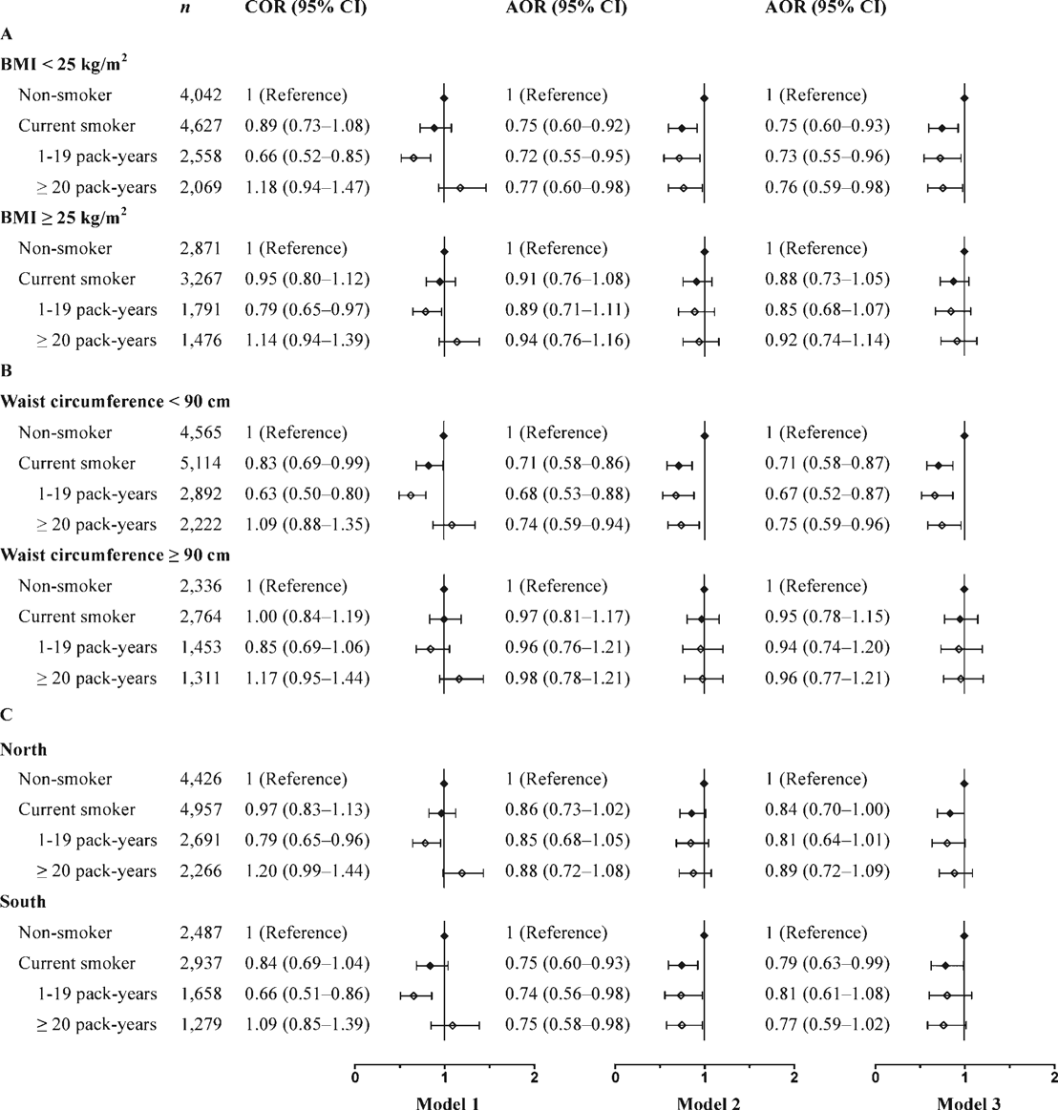
Association between smoking exposure and NDM according to BMI, waist circumference, or region strata. (A) Associations between smoking exposure and NDM in Chinese males stratified by BMI category. (B) Associations between smoking exposure and NDM in Chinese males stratified by waist circumference category. (C) Associations between smoking exposure and NDM in Chinese males stratified by region category. Model 1: Without adjusted variables. Model 2: Adjusted for area of residence (northern/southern and urban/rural in Fig 1A and Fig 1B; and urban/rural in Fig 1C), age, alcohol consumption status, physical activity, education levels and family history of diabetes. Model 3: Adjusted for waist circumference, TG, HDL-C, and SBP in addition to the factors in Model 2. Abbreviations: AOR: adjusted odds ratio; COR: crude odds ratio; HDL-C: high-density lipoprotein cholesterol; NDM: newly-diagnosed diabetes mellitus; SBP: systolic blood pressure; TG: triglycerides.

In Model 2, waist circumference- and BMI-stratified analysis showed that the negative associations between smoking exposure (smoking status, pack-years) and NDM were significant in males of a normal weight but not in males who were overweight/obese or who had central obesity. The corresponding AORs for current smokers with 1–19 and ≥ 20 pack-years were 0.72–0.77 in the group without general obesity and 0.68–0.74 in the group without central obesity (all *P* < 0.05). Additional adjustment did not alter the negative associations between smoking exposure and NDM in Model 3 (Fig 1A and Fig 1B).

There was also a regional difference in the association between smoking exposure and the likelihood of diabetes. Among men living in southern China, current smokers, light smokers (1–19 pack-years) and heavy smokers (≥ 20 pack-years) were significantly less likely to have NDM; the corresponding AORs were 0.75, 0.74 and 0.75, respectively (all *P* < 0.05); however, these negative associations were not observed among males living in northern China. The associations between smoking exposure (except current smoking) and NDM (observed in Model 2) almost disappeared in males living in southern China after adjusting for the confounding factors of the metabolic indices in Model 3 (Fig 1C).

### Association between smoking exposure and other metabolic disorders in males of a normal weight and males who were overweight or obese (Table 4)

Using non-smokers as the reference group, multivariable regression analysis showed that current smokers were less likely to have central obesity, elevated BP, and JCDCG-MetS (AOR: 0.82, 0.74, and 0.86, all *P* < 0.05) among males of a normal BMI, while current smokers were more likely to have central obesity and reduced HDL-C (AOR: 1.14 and 1.25, *P* < 0.01) among males who were overweight/obese. There were negative linear relationships of pack-years of smoking to elevated TG and elevated BP (*P* = 0.009 and *P* < 0.001, respectively, data not shown in Table 4), and the respective AORs ranging from 0.95 to 0.84 and 0.75 to 0.72 for 1–19 pack-years and ≥ 20 pack-years among men with a normal weight. However, there was a positive linear relationship between pack-years of smoking and reduced HDL-C (*P* < 0.001, data not shown in Table 4), and the AORs ranging from 1.11 to 1.43 for 1–19 pack-years and ≥ 20 pack-years (all *P* < 0.04) in males who were overweight/obesity. Additionally, there were no significant associations between quitting smoking and any metabolic disorder either among males of a normal weight or males who were overweight or obese (Table 4).

**Table 4:** Association between smoking exposure and other metabolic disorders among males with a normal weight and males who were overweight or obese.

Subgroup	N	Normal weight		N	Overweight or Obesity	
		Adjusted OR^a^	*P*-value		Adjusted OR^a^	*P*-value
**Smoking status**						
Central obesity						
Non-smoker	4,001	1 (Reference)	0.002	2,843	1 (Reference)	0.081
Ex-smoker	685	1.19 (0.94–1.51)	0.143	763	1.09 (0.91–1.32)	0.338
Current smoker	4,540	0.82 (0.71–0.96)	0.011	3,219	1.14 (1.02–1.28)	0.026
Elevated TG						
Non-smoker	3,993	1 (Reference)	0.166	2,834	1 (Reference)	0.256
Ex-smoker	682	0.91 (0.76–1.10)	0.352	762	1.10 (0.93–1.29)	0.265
Current smoker	4,528	0.91 (0.82–1.01)	0.063	3,201	1.09 (0.98–1.21)	0.127
Reduced HDL-C						
Non-smoker	3,964	1 (Reference)	0.454	2,794	1 (Reference)	0.001
Ex-smoker	672	1.01 (0.82–1.25)	0.910	754	1.01 (0.83–1.23)	0.921
Current smoker	4,479	1.07 (0.96–1.20)	0.220	3,178	1.25 (1.11–1.41)	< 0.001
Elevated BP						
Non-smoker	4,007	1 (Reference)	0.001	2,838	1 (Reference)	0.197
Ex-smoker	684	1.02 (0.86–1.22)	0.795	765	1.09 (0.91–1.32)	0.341
Current smoker	4,542	0.74 (0.67–0.82)	< 0.001	3,218	0.94 (0.84–1.05)	0.259
Metabolic syndrome						
Non-smoker	3,940	1 (Reference)	0.144	2,772	1 (Reference)	0.588
Ex-smoker	669	0.92 (0.72–1.18)	0.527	749	1.05 (0.89–1.24)	0.573
Current smoker	4,437	0.86 (0.74–0.999)	0.049	3,153	1.06 (0.95–1.18)	0.316
**Pack-years**						
Central obesity						
Non-smoker	4,001	1 (Reference)	0.052	2,843	1 (Reference)	0.093
1–19 pack-years	2,510	0.81 (0.68–0.98)	0.027	1,765	1.14 (0.997–1.31)	0.056
≥ 20 pack-years	2,030	0.85 (0.71–1.02)	0.079	1,454	1.13 (0.98–1.31)	0.103
Elevated TG						
Non-smoker	3,993	1 (Reference)	0.042	2,834	1 (Reference)	0.255
1–19 pack-years	2,503	0.95 (0.84–1.07)	0.416	1,754	1.11 (0.98–1.26)	0.101
≥ 20 pack-years	2,025	0.84 (0.74–0.96)	0.012	1,447	1.05 (0.92–1.21)	0.436
Reduced HDL-C						
Non-smoker	3,964	1 (Reference)	0.476	2,794	1 (Reference)	< 0.001
1–19 pack-years	2,483	1.08 (0.95–1.23)	0.231	1,750	1.11 (0.97–1.28)	0.138
≥ 20 pack-years	1,996	1.05 (0.91–1.22)	0.514	1,428	1.43 (1.23–1.67)	< 0.001
Elevated BP						
Non-smoker	4,007	1 (Reference)	< 0.001	2,838	1 (Reference)	0.463
1–19 pack-years	2,506	0.75 (0.66–0.84)	< 0.001	1,764	0.95 (0.83–1.08)	0.408
≥ 20 pack-years	2,036	0.72 (0.64–0.82)	< 0.001	1,454	0.92 (0.79–1.06)	0.246
Metabolic syndrome						
Non-smoker	3,940	1 (Reference)	0.210	2,772	1 (Reference)	0.612
1–19 pack-years	2,464	0.88 (0.73–1.06)	0.190	1,736	1.05 (0.93–1.20)	0.418
≥ 20 pack-years	1,973	0.86 (0.72–1.03)	0.110	1,417	1.06 (0.92–1.21)	0.410

^a^Adjusted for area of residence (northern/southern, urban/rural), age, alcohol consumption status, physical activity, and education level

Abbreviations: BP: blood pressure, HDL-C: high-density lipoprotein cholesterol, TG: triglycerides

## Discussion

Although smoking has been known as the most leading preventable cause of death and disease worldwide [1], it remains unclear as to how smoking is related to the risk of developing diabetes. One key insight that could help to delineate the relationship is how to interpret the results of epidemiological studies. As a result of weak associations, the reliance on self-reporting information regarding smoking exposure, and the impossibility of fully adjusting for all confounders, the methodological shortcomings of epidemiology in exploring the association between smoking and diabetes become more serious and could yield false results. Even sophisticated statistical analyses and increasing the size of a study will never overcome the deficiencies of such datasets [21]. Given the significant heterogeneity across studies and the difference in study population characteristics, study quality, and analysis strategies, the combined conclusion drawn from the meta-analysis may be not appropriate. Epidemiological investigations can still only provide suggestive evidence of a causal relationship. More attention should be paid on how to improve the quality of such studies, how to seek the more objective indices of exposure assessment, and how to conduct the analysis.

The negative association between smoking and hyperglycemia was initially observed in a region-, sex-, and age-matched case–control study (6,909 prediabetes–NGR and 2,867 NDM–NGR groups) based on the same dataset. This finding was against our understanding and prompted us to conduct this current study. Although the most researchers consider that smoking increases the risk of diabetes, some data support the reverse association: (1) there is biological plausibility of the exposure-disease relationship support; for example, smoking does indeed causally decrease weight [22] and subsequently decrease diabetes risk. (2) Newly quitting smoking is associated with higher risk of developing diabetes [23] and a deterioration in the glycemic control of patients with type 2 diabetes [24].

In this study, we excluded individuals with a BMI below 18.5 kg/m^2^ to eliminate the effect of cachexia on the relationship between smoking and diabetes. Since it has been proven that a longer duration of cigarette smoking influences BMI/waist circumference [4, 25, 26], we mainly discussed the results, only adjusting for demographic and behavioral indices, except where specifically noted. The main findings were as follows. (1) Generally, the respective likelihoods of prediabetes and diabetes as screened by the OGTT were 9% and 20% lower for current smokers than non-smokers. (2) Furthermore, significant negative associations between smoking and NDM existed in males of a normal weight (BMI < 25 kg/m^2^: AOR = 0.75, *P* = 0.007; waist circumference < 90 cm: AOR = 0.71, *P* = 0.001) and males living in southern China (AOR = 0.75, *P* = 0.009), but not in males who were overweight/obese, males with central obesity, or males living in northern China. (3) Among males of a normal BMI, compared to non-smokers, current smokers were less likely to be centrally obese and have elevated BP (AOR: 0.82 and 0.74, both *P* < 0.05), and heavy smokers (≥ 20 pack-years) were less likely to have elevated TG (AOR = 0.84, *P* = 0.012). (4) There was no association between quitting smoking and metabolic disorders in either males of a normal weight or males who were overweight/obese.

Although most studies have reported a positive association between smoking and MetS [27], others did not observe associations between smoking and elevated FPG, elevated BP or central obesity [28, 29]. Population-based observation studies have drawn inconsistent and even controversial conclusions on the link between cigarette smoking and MetS components [10, 28, 29]. In this study, current smokers were less likely to be centrally obese or have elevated BP among males of a normal BMI, but were more likely to be centrally obese or have reduced HDL-C than non-smokers among males who were overweight or obese (all *P* < 0.05), similar findings in smokers have reported in other studies [30, 31]. Furthermore, male smokers who had smoked ≥ 20 pack-years had more favorable metabolic profiles, such as a lower likelihood of being centrally obese, an elevated TG, or an elevated BP, than non-smokers among males of a normal BMI. These results hint that smoking may reduce the development of diabetes in Chinese males with a normal weight partly by mediating beneficial metabolic profiles, as metabolic disorders are established risk factors for diabetes. Our results, from another prospective, supported the viewpoint that overweight and obesity is an underlying driver in the development of type 2 diabetes [32]. Onat et al. also noted that the significantly lower frequency of baseline abdominal obesity and lower BP in heavy smokers mediated the “protective” effect of smoking on MetS and diabetes in Turkish females [10].

Smoking increases energy expenditure [33, 34] and suppresses appetite [22], which may lead to a lower BMI/smaller waist and subsequently, a lower prevalence of diabetes in current smokers. The effects of cigarette smoking on glucose metabolism and insulin resistance are still disputed. This study showed that current smokers had lower 2-h PG, which is supported in part by a previous study [35, 36] and a meta-analysis which concluded that current smokers had comparable FPG and lower 2-h PG than never-smokers [12]. Although some studies have indicated that smoking may reduce insulin sensitivity and enhance insulin resistance [37, 38], on the contrary, a small number of other studies showed that cigarette smoking was not associated with insulin resistance [36, 39] and even improved insulin sensitivity in female smokers [40]. This analysis showed that, compared to non-smokers, light and heavy smokers had a lower median HOMA-IR (mU·mmol/L^2^; 1.60, 1.46 vs. 1.71, both *P* < 0.05) and higher median ISI_Gutt_ (mg·L^2^/[mmol·mU·min]; 95.68, 90.84 vs. 88.35, both *P* < 0.05; data not shown in Results).

In addition, our results suggest that the weak effect of smoking predominates in men with a lower BMI/smaller waist. The effects of smoking may not only influence BMI, but may also depend on BMI, as noted by previous studies [5, 11] and discussed below.

Among males of a normal weight, both light and heavy current smokers were significantly less likely to be diagnosed with diabetes than non-smokers (AOR 0.68–0.77, all *P* < 0.05), but this effect was not observed among males who were generally overweight/obese or had central obesity. Other studies also observed that the strength and direction of the association between smoking and diabetes differed as BMI/waist circumference varied [5, 11]. A prospective study observed that light smoking reduced the risk of type 2 diabetes in lean males, but that heavy smoking increased the risk in obese males [11]. In addition, the InterAct Consortium stated that the positive association between smoking and incident diabetes, modified by BMI and waist circumference, was slightly stronger in males with a normal weight than in those with overall adiposity among current smokers [5]. In this study, the interactions analysis showed significant negative associations between smoking and NDM in males with a normal weight, but no associations in males who were overweight/obese or males with central obesity. These results showed that when studying the association between diabetes and smoking, the interaction between smoking and weight category should be taken into consideration. At the same time, the negative association was more obvious in males living in southern China than in northern China. We noted obvious differences in obesity characteristics between the northern and southern populations based on the same dataset: the latter had smaller waists and lower BMI, with an approximately 4 cm difference in waist circumference and a 1 kg/m^2^ difference in BMI between the males living in southern and northern China (unpublished data). Body weight partly modifies the regional differences observed in the impact of smoking on NDM.

The reasons that our results differ from those of previous studies can potentially be explained by the following observations. (1) There are obvious differences in anthropometry and glucose metabolism characteristics between the Chinese population and white populations. For example, the Chinese population has a significantly lower BMI /waist circumference and higher risk of diabetes at a given BMI or waist circumference than white populations [41, 42], and Chinese are more likely to have impaired glucose tolerance [2] while white people tend to have impaired fasting glucose [43]. (2) The use of different diabetes diagnostic methods could also be significant. We used a standard OGTT while some researchers adopted self-reported data or medical care records [5, 44, 45]. (3) The association between smoking and diabetes was adjusted by different confounding factors. Smokers tend to lose weight in comparison with non-smokers [22], and lower weight is protective against diabetes and improves insulin sensitivity. Our study demonstrated a slightly lower age-adjusted mean of BMI (kg/m^2^) in current smokers compared to non-smokers (24.3 vs. 24.5, *P* < 0.001; data not shown in Results) among Chinese adults with a BMI ≥ 18.5 kg/m^2^. Assuming the above premise is accepted, then the adjustment for BMI may result in a population bias [46].

Also, although there are several previous studies in Chinese men associating diabetes risk with smoking, some limitations may affect their conclusions’ validity to some extent. (1) In one study, all cases with type 2 diabetes were identified according to the participants’ self-reports during two surveys which means that a considerable proportion of cases may not be detected (60.7% cases as reported in Reference [2]) and may lead to a major misclassification bias [47]. (2) In another study, participants were selected from a specific population, which made the result difficult to generalize, and smoking was defined as current cigarette smoking or ex-smoking [48]. (3) Zhu et al. reported a non-significant association between smoking and elevated glycemia, OR (95% CI) being 0.93 (0.527–1.64), based on a retrospective study with a smaller sample and a lower follow-up duration [49]. Additionally, some cohorts did not appear to specifically be designed to explore the association between smoking and diabetes risk according to the selection criteria of the study population at the baseline, which can affect statistical conclusion validity to some degree.

Some population-based studies have indicated a sex difference in the relationship between smoking exposure and diabetes [44, 50]. Most studies focusing on male adults showed a positive association between smoking and diabetes [5, 45], while studies of females were more contentious, and even showed a weaker negative association [5, 10, 51]. Considering the sex differences in terms of the proportion and strength of smoking exposure, and the relationship between diabetes and smoking status, we explored this issue separately for males and females in two manuscripts. We found that Chinese females have a significantly lower rate (2.0%) of tobacco use than Chinese males (48.5%). Smoking was negatively associated with hyperglycemia and prediabetes alone, but not NDM. The corresponding adjusted ORs (95%CIs) for hyperglycemia, prediabetes alone, and NDM were 0.69 (0.57–0.83), 0.64 (0.52–0.80), and 0.79 (0.59–1.06), respectively (unpublished data). Current female smokers were less likely to have general overweight/obesity, elevated blood pressure, and metabolic syndrome than non-smokers were (all *P* < 0.05) (unpublished data).

As smoking is an important risk factor for other chronic diseases such as cancer, lung disease and cardiovascular disease, and remains the leading cause of preventable deaths worldwide [1], smokers should still be encouraged to stop smoking to improve their quality of life.

### Study strengths and weaknesses

Detailed information on smoking and complete assessment of metabolic indices allowed us to perform this large-scale nationwide case-control study. We focused on newly diagnosed cases and analyzed metabolic disorder profiles among subpopulations with different smoking exposure levels, which may aid our understanding of the possible mechanism of smoking in the development of diabetes. We noted that measures of association are most often evaluated by using regression analysis. Regression adjustment is a form of stratification, which most often relies on large sample size for inference [46]. Therefore, enough sample sizes are necessary to provide sufficient statistical power. In this study, the large sample size helps us find the weak association between smoking and diabetes.

Our study is a cross-sectional design, and thus a causal relationship was not obtained. Changes in metabolic disorder profiles may occur in individuals with a long history of smoking. In addition, a major limitation of this case-control study is its susceptibility to recall bias. Data collected by questionnaire depended on memory of past smoking exposure. The exposure levels may be misclassified.

## Conclusions

Glucose concentrations were significantly lower at fasting and 120 min in current smokers than non-smokers. When diabetes was screened using FPG and 2-h PG, current smokers had a 9% and 20% lower likelihood of prediabetes and diabetes, respectively, than non-smokers. Furthermore, the negative association between smoking and NDM was more significant in males with a lower BMI/smaller waist. The association between smoking, body fat, and incident diabetes in different ethnic groups needs further investigation.

## Supporting Information

S1 Appendix Members of the China National Diabetes and Metabolic Disorders Study Group. (DOC)
