# Supplementary material for: Cigarette Smoking Is Associated with a Lower Prevalence of Newly Diagnosed Diabetes Screened by OGTT than Non-Smoking in Chinese Men with Normal Weight
Source: PLoS One. 2016 Mar 8;11(3):e0149234. doi: 10.1371/journal.pone.0149234 (PMC4783042; doi:10.1371/journal.pone.0149234)
Supplement: S1 Appendix — (DOC) [file pone.0149234.s001.doc]

**S1 Appendix 1. The China National Diabetes and Metabolic Disorders Study Group**

Consulting Members: Kunsan Xiang, Jialun Chen, Changyu Pan, and Zuzhi Fu.

Study Group Field Centers (all field centers contributed to this work equally):

China-Japan Friendship Hospital, Beijing, China: Wenying Yang (principal investigator), Jianzhong Xiao, Zhaojun Yang, Danjie Ruan (Huairou first Hospital), Yufeng Li (Pinggu Hospital), Shi Bu, Hongliang Li, Peng Wang, Xueli Liu, and Zhiyun Xiao.

Chinese People’s Liberation Army General Hospital, Beijing, China: Juming Lu (principal investigator), Jingtao Dou, Nan Jin, Jing Li, Shuyu Wang, Baojing Zhang, Liguang Dong.

The Third Affiliated Hospital of Sun Yat-sen University, Guangzhou, China: Jianping Weng (principal investigator), Longyi Zeng, Panwei Mu, Yanhua Zhang, Qiuqiong Yu, Keyi Lin, Yan Sun, and Xiaodong Xu.

Shanghai Jiao Tong University Affiliated Sixth People’s Hospital, Shanghai, China: Weiping Jia (principal investigator), Xuhong Hou, Xiaojing Ma, Huijuan Lu, Dajing Zou (Changhai Hospital), Renming Hu (Huashan Hospital), Huilin Gu (Huayang Community Health Center), Yuhua Yao (Linfen Community Health Center), Weizhen Shen (Pengpu Community Health Center), Junbin Huang (Tianmu Community Health Center), and Zhijian Pan (Anting Community Health Center)

Peking University People’s Hospital, Beijing, China: Linong Ji (principal investigator), Xianghai Zhou, Xueyao Han, Xiuying Zhang, Yingying Luo, Xiuqing Sun, Dong Zhao (Luhe Hospital), Huifang Xing (Mentougou Hospital), Lei Liu (Haidianqu Hospital), and Xinquan Zhang (Jingyuan Hospital)

The First Affiliated Hospital of China Medical University, Liaoning, China: Zhongyan Shan (principal investigator), Yaxin Lai, Xiaochun Teng, Ling Shan, Jiani Wang, Liangfeng Shi, Sen Wang, Li Lu, Fengwei Jiang, and Beibei Wang.

Shanxi Provincial People’s Hospital, Shanxi, China: Jie Liu (principal investigator), Ling Hu, and Yuying Hou (Shan Xi Medical University).

West China Hospital, Sichuan University, Sichuan, China: Haoming Tian (principal investigator), Xingwu Ran, Yan Ren, and Hongling Yu; the First People’s Hospital of Liangshan Yi Nationality Autonomy District, Xichan City (Lisheng Cao, Hualin Lu, Xiaohua Xie); Yulin Community Health Center of Wuhou District, Chengdu (Xilian Gao); and the First Hospital of Longquyi District, Chengdu (Zhong Li).

Xijing Hospital, The Fourth Military Medical University, Shanxi, China: Qiuhe Ji (principal investigator), Fei Chen, and Yaping Zhang.

The Affiliated Drum Tower Hospital of Nanjing University Medical School, Jiangsu, China: Dalong Zhu (principal investigator), Yun Hu, Guoyu Tong, Ning Xu, Jinluo Cheng, Junjian Chen, Fei Wang, Jiong Pei.

People’s Hospital of Xinjiang Uygur Autonomous Region, Xingjiang, China: Jiapu Ge (principal investigator), Wan Yi, Rong Wang (Bayi Steel’s Hospital), Gang Han, Huijie Mu (Habahe County Hospital), and Maimaiti Aireti ( Moyu County Hospital).

Fujian Provincial Hospital, Fujian, China: Lixiang Lin (principal investigator), Gang Chen, Jingxin Zhao, Shuyu Yang, and Mingzhu Lin (Xiamen First Hospital), Yadong Zhang, Fengyuan Zhu (Sanming First Hosptial), Mei Tu (Longyan First Hospital), Shanghua Xu (Nanping First Hospital), Weihong Lin (Jiangle County Hospital).

Qilu Hospital of Shandong University, Shandong, China: Li Chen (principal investigator), Yu Sun, Xiaolin Dong (Jinan Central Hospital), Yiling Fu, Kehua Zhou, Jinbo Liu, Zhenzuo Li, Yuxin Xu, Peng Lin, and Wenjuan Li.

Peking University First Hospital, Beijing, China: Xiaohui Guo (principal investigator), Junqing Zhang, Aimei Dong, Dongming Huang, Changchun Xue (Daxinqu Hospital), and Junqing Liu (Shichahai Community Health Center).

Henan Provincial People’s Hospital Henan, China: Zhigang Zhao (principal investigator), Guijun Qin, Yong Yan, Peiyu Yao, and Qinchu Li.

The Second Affiliated Hospital of Harbin Medical University, Heilongjiang, China: Qiang Li (principal investigator), Kaiting Chen, Nannan Wu, Yan Feng, Xiaoying Liu, and Guozhong Li.

The Second Xiangya Hospital of Central South University, Hunan, China: Zhiguang Zhou (principal investigator), Weili Tang, Qiong Feng, Yuju Qin, Xinwen Qiu, Diaoxiang Xiao, Aiping Qin, Bin Liao, Zifang Gao, Liang Xiang.
